# A Needs-Based Support for #MeToo: Power and Morality Needs Shape Women’s and Men’s Support of the Campaign

**DOI:** 10.3389/fpsyg.2020.00593

**Published:** 2020-03-31

**Authors:** Anna Kende, Boglárka Nyúl, Nóra Anna Lantos, Márton Hadarics, Diana Petlitski, Judith Kehl, Nurit Shnabel

**Affiliations:** ^1^Department of Social Psychology, ELTE Eötvös Loránd University, Budapest, Hungary; ^2^Doctoral School of Psychology, ELTE Eötvös Loránd University, Budapest, Hungary; ^3^The School of Psychological Sciences, Tel Aviv University, Tel Aviv, Israel; ^4^Department of Psychology, University of Osnabrück, Osnabrück, Germany

**Keywords:** #MeToo, collective action, gender equality, needs-based model, sexual harassment

## Abstract

The #MeToo campaign mobilized millions of women around the world to draw attention to the pervasiveness of sexual harassment. We conducted an online survey in Hungary (*N* = 10,293) immediately at the campaign’s onset, and two subsequent studies in Israel and Germany (*N*s = 356, 413) after it peaked, to reveal the motivations underlying people’s support for, or criticism of the campaign. Integrating the assumptions of the needs-based model of reconciliation and system justification theory, we predicted and found that, in all three samples, lower gender system justification was associated with (a) women’s perception of the campaign as empowering, and men’s (b) higher perception of the campaign as an opportunity for moral improvement, and (c) lower perception of the campaign as wrongfully staining men’s reputation. As expected, in all three samples, (a) perceptions of the campaign as empowering among women, and an opportunity for moral improvement among men, were associated with greater campaign support, whereas (b) men’s perceptions of the campaign as wrongfully staining their moral reputation were associated with lower campaign support. Thus, the link between system justification and campaign support was mediated by women’s empowerment needs, and men’s morality-related needs. In addition, perceptions of the campaign as disempowering their ingroup (i.e., presenting a status threat) predicted reduced campaign support among men in the Hungarian and Israeli samples, but not the German sample. We discuss the practical implications of these results for gender equality movements in general, and sexual harassment in particular, by identifying the psychological obstacles and catalysts of women’s and men’s support for social change.

## Introduction

The phrase “Me Too” was coined by Tarana Burke, an African American civil rights activist, who began using it in 2006 to raise awareness about the pervasiveness of sexual abuse and assault in society. When actress Alyssa Milano used the hashtag #MeToo in a Twitter post in October 2017, she had a similar goal in mind: emphasizing the structural aspect of sexual harassment embedded in gender relations in society. The #MeToo campaign against sexual harassment went viral globally. The campaign was successful in terms of awareness raising, actual charges against perpetrators of sexual assaults, and support for victims to come forward with their personal stories (Rhodan, 2018; Seales, 2018). Nevertheless, #MeToo was also heavily criticized, suggesting that the campaign hampers due process, might increase false accusations (Piacenza, 2018), fails to distinguish between rape and harassment, and collectively blames all men and victimizes all women (Arceneaux, 2018; O’Connell, 2018).

The purpose of the present research was to examine the psychological motivations that underlie peoples’ support for, or opposition to the campaign. One thing in common for both supporters and opponents is that they evaluated #MeToo through a gendered perspective on sexual harassment, embracing or criticizing the fact that, within this campaign, women appeared as collective victims of sexual harassment, implying that men were the collective perpetrators. We therefore reasoned that the theoretical framework of the needs-based model (Shnabel and Nadler, 2015), according to which victims and perpetrators have different power- and morality-related psychological needs, can be of relevance for understanding the motivations underlying campaign support. Recent research within the framework of the needs-based model (Hässler et al., 2019) has shown that women’s and men’s power- and morality-related needs, resulting from the perception of their ingroup as a victim or a perpetrator group, are influenced by their system justification motivation (i.e., the motivation to accept and legitimize the societal *status quo*, Jost and van der Toorn, 2012). Thus, we examined whether and how participants’ gender system justification (i.e., the motivation to justify existing gender arrangements) predicts their perception of the campaign as addressing their power and morality-related needs which, in turn, determines their support of or opposition to the #MeToo campaign.

### Group Members’ Needs for Empowerment and Morality: The Perspective of the Needs-Based Model

The main tenet of the needs-based model is that following transgressions, victims and perpetrators experience different psychological needs (Shnabel et al., 2009). Members of victim groups feel weak and disrespected, and therefore experience a need for empowerment: they wish to enjoy a better status and have more influence in society. In contrast, members of perpetrator groups experience a threat to their ingroup’s moral reputation and are motivated to restore their positive moral identity. This motivation can manifest in two distinct forms (Allpress et al., 2014): perpetrators may experience *essence shame* due to the violation of moral values, and consequently wish their ingroup to acknowledge its culpability and behave more morally. Alternatively, they may experience *image shame*, which signifies the defensive need for restoring their ingroup’s moral reputation, without changing its moral conduct (e.g., by having outgroup members acknowledge that they do receive fair treatment).

Whereas earlier research examined the needs-based model in contexts of direct violence, in which the roles of “victim” and “perpetrator” groups is consensual and clear-cut (e.g., the Holocaust; Shnabel et al., 2009), subsequent research examined it in contexts of so-called ‘structural violence’ (i.e., group-based inequality, Galtung, 1969), which is characterized by ambiguity with regard to the advantaged group’s “culpability.” Aydin A. L. et al. (2019) revealed that the psychological needs of advantaged and disadvantaged group members (e.g., members of higher and lower social classes; Aydin A. et al., 2019) correspond to those of victims and perpetrators. Whereas disadvantaged group members experience threat to their status and identity as competent and are therefore motivated to gain respect (Bergsieker et al., 2010), advantaged group members experience threat to their moral identity (e.g., they may be perceived as prejudiced and bigoted, Fiske et al., 2002) and therefore seek moral-social acceptance (Bergsieker et al., 2010). Furthermore, whereas disadvantaged group members were found more willing to engage in collective action toward equality following an empowering, competence-reassuring message from their outgroup, advantaged group members were more willing to engage in social change action following an accepting message that reassured their moral identity (Shnabel et al., 2013). Further evidence that concerns about their moral identity play a critical role in determining advantaged group members’ support of social movements comes from research findings that advantaged group members’ support for different forms of collective action was primarily influenced by the extent to which these actions affect their ingroup’s image as moral (which was even more important for them than the actual effectiveness of these actions in reducing inequality, Teixeira et al., 2019).

However, besides group affiliation (i.e., advantaged vs. disadvantaged) the experience of power and morality-related needs also depends on the extent to which group-based disparities are perceived as legitimate or illegitimate (Siem et al., 2013). In the particular context of gender relations, in response to information about group-based inequality and societal discrimination against women, women reported a higher need for power (e.g., wish that their ingroup would have more influence in society) compared to men (Hässler et al., 2019; Study 2). However, women’s and men’s power needs also depended on their motivation to justify the gender system, such that system justification predicted a lower need for power among women and higher need for power among men. In terms of the need to restore the ingroup’s moral essence, compared to women, men reported more moral shame and wish that their ingroup would act more morally toward the outgroup. System justification was negatively related to men’s wish to restore their ingroup’s moral essence (e.g., men who were high on system justification reported less moral shame), yet it was unrelated to women’s need for moral essence. Also, system justification was positively related to men’s wish to defend their ingroup’s moral reputation (e.g., men who were high on system justification wished women to acknowledge that they receive fair treatment from men), yet it was unrelated to women’s need to defend their moral reputation. Besides the potential threat to their moral identity, the societal debate about gender inequality might threaten men’s status. Studies have shown that social movements of advantaged groups (e.g., conservative movements or men’s rights movements) often demand the restoration of their rights, because they experience threats to their status and feel victimized (e.g., Blee and Creasap, 2010).

Based on these findings, we reasoned that the needs-based model may be applicable to the context of sexual harassment in general and the #MeToo campaign in particular, in which the groups of women and men were associated with victim and perpetrator groups. The relevance of a needs-based approach for the #MeToo was echoed in accompanying viral hashtags: reflecting women’s need for empowerment, #TimesUp suggested that women should take more action against sexual harassment; #HowIWillChange (see Vagianos, 2017) suggested that men could consider the movement as an opportunity to show moral improvement; and #NotAllMen (initiated earlier, but resurfaced in this context) became a counter-campaign to reject accusations that stains the moral reputation of men. In addition, because the #MeToo campaign has questioned the structural inequalities of gender relations, and consistent with Hässler et al.’s (2019) findings, we expected women’s and men’s system justification motivation to determine their power and morality-related needs, their resulting perceptions of the campaign of addressing or threatening these needs, and consequent support for, or opposition to the campaign.

### System Justification Shapes People’s View of Gender Equality Movements

The original statement of the #MeToo campaign – *“If all the women who have been sexually harassed or assaulted wrote ‘Me Too’ as a status, we might give people a sense of the magnitude of the problem”* – pointed to the notion that sexual harassment, although realized mostly in interpersonal encounters, is a group-based grievance of women (Tangri et al., 1982; Wayne, 2000). Group-based grievances and the perception of injustice can contribute to the development of a politicized collective identity of group members, and to engagement in the struggle to change existing intergroup relations (see Simon and Klandermans, 2001). The #MeToo campaign was therefore a case of collective action mobilizing women to raise awareness about the phenomenon of sexual harassment and change the *status quo* of existing social arrangements.

However, not all women, and certainly not all men, are ready to change the *status quo*. According to system justification theory (Jost and Banaji, 1994), the perception of the social system as legitimate satisfies basic epistemic, existential, and relational needs. Therefore, people are motivated “to defend, justify, and bolster aspects of the *status quo* including existing social, economic, and political institutions and arrangements” (Jost and van der Toorn, 2012, p. 334), even if their own ingroup suffers from these arrangements. The tendency to justify existing social arrangements may be particularly strong in the context of gender relations (as compared to other contexts of intergroup relations, such as the relations between different racial, religious, or ethnic groups). One reason is that the gender status asymmetry is universally present in all societies, and therefore, seem inevitable (Sidanius and Pratto, 2001). Moreover, the relations between men and women are characterized by high interdependence in a social, economic, and emotional sense due to reproductive needs (Guttentag and Secord, 1983), and the cultural histories of human societies creating socio-economic interdependence (Wood and Eagly, 2002). Consequently, both women and men are motivated to maintain harmonious relations, and avoid open conflict (Jackman, 1994). This motivation is an obstacle to social change because when intergroup relations are characterized by a desire for harmony, people make efforts to maintain social cohesion, while hindering the motivation to expose group-based inequality and engage in collective action for changing it (Wright and Lubensky, 2009). This process has been observed in various contexts, including the one of the #MeToo campaign (Kunst et al., 2018).

While it may be difficult for women to perceive their own disadvantages within gender relations due to the motivation to justify the system, it may be even harder for men to recognize these inequalities and get involved as allies. Naturally, men have fewer chances to get first-hand experience of gender-based inequalities in general and sexual harassment in particular. Moreover, as members of the advantaged group, they are less likely to recognize their own privileges (Becker and Barreto, 2014) both because advantaged group members are generally motivated to uphold the *status quo* and disregard information challenging their social status (Leach et al., 2002) and because criticism of unearned privileges may appear as a threat to their moral standing (Maass et al., 2003). Men’s engagement in the struggle against sexual harassment is therefore dependent on their moral convictions (van Zomeren et al., 2011) and efforts to improve their own moral reputation (Hopkins et al., 2007) – both of which are hindered by men’s tendency to justify the existing system.

Thus, in the present research we predicted that people’s general tendency to justify the existing social system regarding gender relations, would be associated with less support for, and more opposition to, the #MeToo campaign (see Kunst et al., 2018). Based on our conceptualization of the #MeToo campaign as a form of collective action, this prediction is consistent with previous findings about the negative association between system justification and collective action tendencies, among both the advantaged and the disadvantaged (Osborne et al., 2019). As explained in the previous section, we further predicted that the effect of system justification on support for (or opposition to) the #MeToo campaign would be mediated by the extent to which the campaign is perceived to address women’s and men’s differential needs for power and morality.

In sum, evidence shows that women are almost exclusively harassed by men, while men can fall victim to both men and women, which means that men are in an overwhelming majority among sexual harassers (European Union Agency for Fundamental Rights, 2014). Nevertheless, we reasoned that, depending on their motivation to justify the existing gender system, some men and women would view themselves as members of perpetrator and victim groups (respectively), whereas others would reject this view. The endorsement or rejection of the social roles of “victims” and “perpetrators” would in turn influence women’s and men’s perception of the campaign as an opportunity for empowerment and moral improvement or as wrongfully accusing all men as perpetrators and weakening and victimizing all women.

### Gender Inequality Is Closely Connected to Attitudes Toward Sexual Harassment

Gender inequality in education, employment, financial status, political representation, the prevalence of sexual harassment and rape, and the perception of violence against women greatly vary across countries (World Economic Forum, 2017; European Institute for Gender Equality, 2019). Apart from historical, cultural, and economic reasons, gender inequality is maintained by attitudes supporting it (Chapleau and Oswald, 2014). Sexual harassment and rape disrupt the harmony ideal between men and women and can draw attention to these inequalities (see Searles, 1995) while gender system justification prevents the recognition of transgressions by men and the gendered characteristic of rape (Chapleau and Oswald, 2014). The paradoxical connection between gender equality and reported rape (i.e., higher reported rape in more equal countries) underlines that in countries with greater inequalities, women are less likely to report rape because of more hostile attitudes to rape victims (Sable et al., 2006). For example, within Europe, reported rape is lowest in Hungary and Greece, the two lowest ranking countries in terms of gender inequality and highest in Sweden which is the highest ranking country (European Union Agency for Fundamental Rights, 2014).

Consequently, people living in countries with more unequal gender relations tend to be more accepting of the gender *status quo* (Glick et al., 2000), consider rape and sexual harassment as a less significant problem (Yamawaki and Tschanz, 2005; Yamawaki, 2007), and therefore less likely to see the connection between sexual harassment and the gender *status quo*. We can assume that the global #MeToo campaign which aimed to address precisely the prevalence of sexual harassment and its connection to gender relations would be differently received in countries with different degrees of gender equality. For this reason, although we conducted our research originally in Hungary, we replicated it using smaller samples in Israel and Germany to increase external validity of our research. These two additional countries have higher gender equality than Hungary according to the Global Gender Gap Index (World Economic Forum, 2017), but they are quite different from one another too. The World Economic Forum ranks countries based on four fields affecting gender equality: economic participation and opportunity, educational attainment, health and survival, and political empowerment. Germany is placed 12th, Israel 44th, and Hungary 103rd in the ranking.

## Research Question and Hypotheses

**FIGURE 1:**
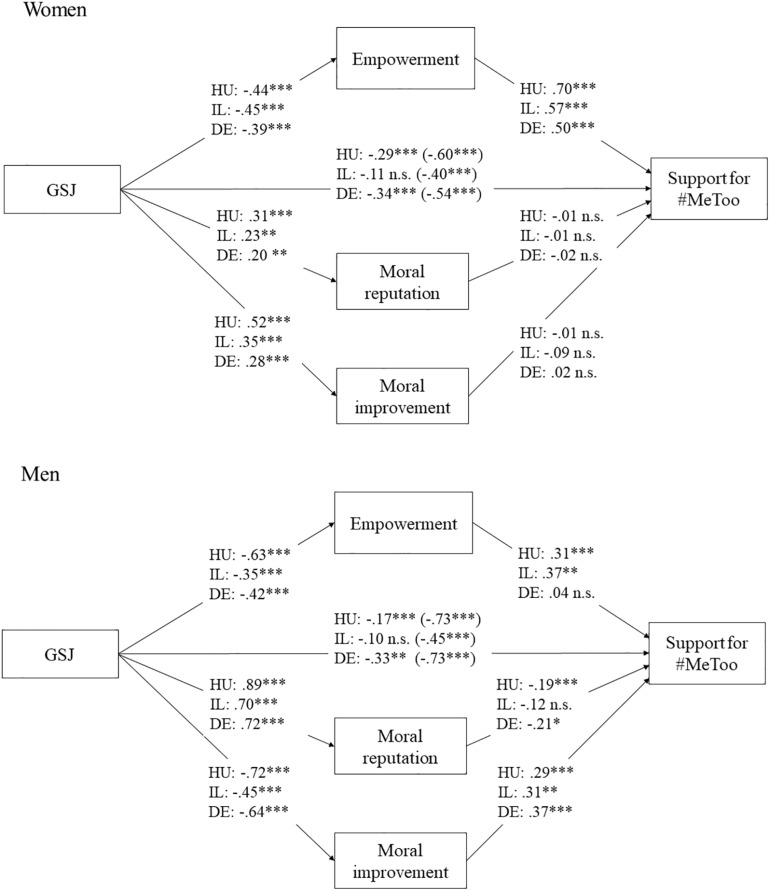
Path-models of support for the #MeToo campaign. Relationship strengths are indicated by unstandardized regression coefficients. ^∗∗∗^*p* < 0.001, ^∗∗^*p* < 0.01, ^∗^*p* < 0.05. HU, Hungarian sample; IL, Israeli sample; DE, German sample.

Our research hypotheses were based on previous theorizing about the connection between gender system justification (similarly to Kunst et al., 2018), and the different psychological needs of members of perpetrator and victim groups. We expected that people with higher gender system justification would support the #MeToo campaign less than those with lower gender system justification, and these effects were expected to be mediated by the perception of the campaign as addressing or thwarting the different needs of men and women as members of perpetrator and victim groups (as visually presented in Figure 1).

In particular, because receiving acknowledgment for the injustice caused to one’s ingroup is a central component of empowerment (Shnabel and Nadler, 2015), we expected that women who are lower on system justification, and generally endorse the view of their ingroup as a victim of unfair disadvantage, would consider the #MeToo campaign as more empowering. Perceptions of the campaign as empowering, in turn, would predict support the campaign. Consistent with Hässler et al.’s (2019) findings that system justification was unrelated to women’s moral needs, we expected that women’s system justification would be unrelated to their perceptions of the campaign as addressing or threatening their moral reputation and the opportunity for moral improvement, and that these morality-related perceptions would not be associated with women’s campaign support. This is because, regardless of the general level of motivation to justify the gender system, women do not see themselves as members of the perpetrator group (or the advantaged group in the context of gender relations).

As for men, we expected that lower gender system justification would predict viewing the campaign as an opportunity to behave more morally toward women, which would in turn predict greater support. On the other hand, higher gender system justification would predict viewing the campaign as wrongfully accusing and morally staining men, predicting less support to the campaign. Based on Hässler et al.’s (2019) findings that system justification predicted men’s greater need for power, we expected system justification to predict men’s perception of the campaign as disempowering for their ingroup (as it might threaten their advantaged status), would in turn translate into reduced campaign support.

Testing these hypotheses has theoretical and practical importance. From a theoretical perspective, the novelty of our research is that we integrate system justification and the different needs of advantaged and disadvantaged groups with support for a social change campaign in the context of gender relations. Previous research has highlighted the relevance of the needs-based model for supporting social change among advantaged and disadvantaged groups (see Shnabel et al., 2013), and the relevance of system justification and the needs-based model for understanding gender relations (see Hässler et al., 2019). However, no research has analyzed how the connection between system justification and support for social change is mediated by the different needs of advantaged and disadvantaged groups so far, and therefore, our findings can shed light on the ways in which social change can become acceptable for groups with different status in society.

From a more practical perspective, understanding the psychological motivations of both men and women in a campaign about gender relations and sexual harassment is quintessential, as social movements benefitting disadvantaged groups are dependent on advantaged group allies to effectively change existing social relations (Thomas et al., 2010). Campaigns against sexual harassment cannot achieve their goals without men’s engagement. Furthermore, our research can contribute to sources for misunderstandings related to the problem of sexual harassment and specifically to the #MeToo campaign. Intergroup misunderstandings constitute a major obstacle to constructive intergroup relations (Demoulin et al., 2009). For example, the fact that men view discussions about sexual harassment through the prism of morality (e.g., wishing to define what constitutes inappropriate behavior toward women), whereas women view such discussions primarily through the prism of empowerment (e.g., wishing to identify ways in which women can gain more control when encountering such behaviors) may be a source of miscommunication. Moreover, identifying the underlying psychological motivations for support or opposition to the #MeToo campaign can help design effective interventions and specific communication strategies to recruit people, men and women, to the struggle against sexual harassment.

## The Present Research

In the wake of the #MeToo campaign, a Hungarian actress came forward with a story of sexual abuse by a theater director, making the global campaign a locally relevant phenomenon. A year after the campaign started, two thirds of the Hungarian population had heard about the campaign (Kovács and Szémann, 2018). Although many previous stories about sexual abuse and rape had been ambiguously presented in the media (Nyúl et al., 2018), following the #MeToo campaign, sexual harassment cases were treated more severely, resulting in harsher consequences for the perpetrators. These results came about despite the fact that Hungary scores poorly on gender equality (World Economic Forum, 2017), and the rate of reporting rape is very low compared to other countries, indicating the pervasiveness of victim blaming attitudes (European Union Agency for Fundamental Rights, 2014; Wirth and Winkler, 2015).

We conducted our first data collection in Hungary, immediately after the #MeToo campaign started. An advantage of this was that it enabled us to recruit a large and diverse sample and tap the initial reactions to the campaign. In order to check the generalizability of our conclusions, we collected more data a few months later in two different cultural contexts: Israel and Germany.

The #MeToo campaign received much attention in these additional two countries too. In Israel, accusations about a local media mogul were made shortly after the launch of the campaign, receiving broad public attention (Lieberman, 2017). In Germany no specific revelations were made about public figures around the peak of the global campaign, therefore the campaign focused on the general phenomenon of sexual harassment and gender equality first. However, in January 2018, accusations against a film director were published in Die Zeit (Simon et al., 2018), which led to responses in support of and against the campaign that were similar to other countries. Although we did not make different predictions across the three contexts, we expected that men with more experience of gender equality would find the campaign less threatening to their position (for a comparison of men’s engagement in gender equality globally, see IPSOS, 2017).

### Participants

We relied on a large convenience sample of *N* = 10,265 in Hungary. Participants were recruited by posting the link of the online questionnaire on Facebook. It was widely shared by individual people and various groups beyond our control. Therefore, we do not have information about the dominant opinions in the groups in which the link was shared. Our call for participants was picked up by online journals, and posted on the websites of *hvg.hu* and *index.hu*, two of the most widely read online news portals in Hungary.

The final sample in Hungary included 3,435 (33.5%) men, and 6,830 (66.5%) women. Respondents had the opportunity not to use the binary distinction to identify their gender but indicate “other” or their wish not to answer, but the questionnaire was designed differently for men and women, therefore these 163 respondents could not complete the questionnaire and were debriefed. The mean age of participants was 36.66 years (*SD* = 12.48). The level of education was higher than average: 70% held university or college degrees, 14.5% were enrolled in a university education at the time of the data collection, and 15.3% had secondary education or lower. For nationality, 97.2% indicated they were Hungarian.

In the two other contexts, we relied on smaller convenience samples (Israel: *N* = 356; Germany: *N* = 413). Data was collected with the help of university students who recruited respondents on social media. Sample sizes were calculated based on the results of the Hungarian data that was collected earlier. Sample size was adequate based on G^∗^Power calculations detecting 95% power for a multiple regression analysis based on the large effect sizes (Faul et al., 2009), but the subsamples of men were below the suggested min. 200 participants for mediation models using scale means (see e.g., Ding et al., 1995). Therefore, these results need to be treated with caution.

There were 132 (37.1%) men, and 222 (62.4%) women in the Israeli sample and 130 (31.5%) men and 283 (68.5%) women in the German sample. The average age of respondents in Israel was *M* = 29.18 years (*SD* = 8.26) and in Germany *M* = 25.99 years (*SD* = 6.23). Most respondents either had a university degree (Israel: 44%; Germany: 43%) or were university students at the time of the data collection (Israel: 49%; Germany: 40%). In the Israeli sample 98.6% indicated that their nationality was Israeli and in the German sample all respondents had a German nationality.

As shown in Table 1, the majority of respondents reported that they did not post any personal stories as part of the #MeToo campaign. Posting own story was highest in Hungary (9.6%; women: 13.2%, men: 2.5%), followed by Israel (5.4%; women: 8.1%, men: 0.8%), finally posting own stories was rather low in the German sample (2.5%, women: 3.6%, men: 0%). There were more respondents who posted or commented in support of the campaign than against it and overall posting in support was over 30% in the samples from Hungary and Israel, and 20% in Germany. In the Hungarian sample women participated more and more positively than men, but there was no difference in the amount of critical posting and comments among men and women in Israel and Germany.

**TABLE 1:** Participation of respondents in the #MeToo campaign by gender.

	**Total (%)**	**Women (%)**	**Men (%)**	**χ*^2^***
**Hungarian sample**				
Posted own story using #MeToo	9.6	13.2	2.5	301.08***
Posted or commented support for #MeToo	32.1	40.4	15.6	632.19***
Posted or commented critique of #MeToo	9.1	7.8	11.5	35.39***
**Israeli sample**				
Posted own story using #MeToo	5.4	8.1	0.8	9.17*
Posted or commented support for #MeToo	34.5	44.1	18.2	24.99***
Posted or commented critique of #MeToo	7.6	9	5.3	1.62
**German sample**				
Posted own story using #MeToo	2.5	3.6	0	4.72
Posted or commented support for #MeToo	20	25.3	8.6	15.45***
Posted or commented critique of #MeToo	4.9	4.3	6.3	1.03

***p < 0.001, *p < 0.05.

### Procedure

The language of the questionnaire was Hungarian, Hebrew, and German corresponding to the national contexts of the data collection. All items were translated to the respective languages from English, and back-translated by an independent translator, unless previously published in the respective language that we indicate when describing the measures of the survey.

We collected data using the online survey platform of Qualtrics. We included all respondents who reached the last question block about the items of the needs-based model. Answers were requested and not forced, but missing data was negligible on all variables used in the analysis. In Hungary, missing data was below 0.3% in the women’s sample, and below 0.1% in the men’s sample, there was no missing data in the Israeli sample and < 0.04% in the German sample.

We collected data between November 2nd and 10th, 2017, in Hungary, between January and September, 2018, in Israel, and between June and October, 2018, in Germany. We report all measures and data exclusions related to the hypotheses of this paper. For exploratory purposes, we measured other variables, but their discussion falls outside the scope of this paper (such as perceived efficacy, rape-myth acceptance, opinion-based identity, inclusiveness of the campaign toward men). Data regarding these additional variables are included in our published dataset at osf.io/rj94d. We conducted the research with the IRB approval of Eötvös Loránd University.

### Measures

We measured *gender system justification* (GSJ) by seven items based on the original 8-item scale of Jost and Kay (2005) adapted and shortened to the context of gender by Hässler et al., 2019, we relied on the German translation for the data collection in Germany). Respondents were requested to express their agreement with the listed items on a 7-point scale from 1 = *completely disagree* to 7 = *completely agree*, as in all other scales of the questionnaire (unless noted otherwise). An example item is “In general, relations between men and women are fair.” (One item “Most policies relating to gender and the sexual division of labor serve the greater good” was omitted from the analysis because of it reduced the internal consistency of the scale to.67 in the Hungarian sample). Information on scale consistencies are shown in Table 3. Mean scores were used in the analysis for gender system justification and all variables in the study. Higher scores represented greater level of system justification beliefs regarding gender relations in society.

Variables measuring the satisfaction (or thwarting) of power- and morality-related needs through the campaign were developed based on previous research about the needs-based model in the context of gender relations (Hässler et al., 2019). This research measured group members’ actual needs, that is, their wish that their ingroup would have more power and influence, their feelings of shame, the wish that their ingroup would behave more morally, and their wish to protect their ingroup’s moral reputation (e.g., by having the outgroup acknowledge that it receives fair treatment from ingroup), reflecting a defensive moral need. Adapting these scales to fit the context of the #MeToo campaign, we measured the extent to which group members felt that the campaign satisfies these power- and morality-related needs; that is, the extent to which the campaign empowers (or disempowers) their ingroup, provides (or fails to provide) the ingroup with an opportunity to behave more morally toward the outgroup, and cleans (or stains) their ingroup’s reputation.

Items were identically phrased for men and women, but the words “men” and “women” were switched for the two gender groups. Perceptions of the campaign as *empowering* the ingroup was measured by four items (e.g., “This campaign empowers women/men”), higher scores represented greater perceived empowerment by the campaign (whereas lower scores represented perceptions of the campaign as weakening one’s ingroup). Perceptions of the campaign as an opportunity for *moral improvement* (stemming from guilt) was measured by three items (e.g., “The campaign makes me wish that women/men would treat men/women in a nicer manner”), relying on previous research that guilt can function as a source of motivation for moral improvement (e.g., Iyer et al., 2003). Higher scores reflected the perception that the campaign was an opportunity for the ingroup to behave more morally toward the outgroup. Perceptions of the campaign as staining the ingroup’s *moral reputation* by four items (e.g., “The campaign unjustifiably stains women’s/men’s moral reputation”). Higher scores indicated that respondents perceived the campaign as more damaging to the moral reputation of the ingroup. Items of the subscales are presented in the Appendix.

Finally, we generated four items to capture *support* intentions related to the #MeToo campaign that were context-specific and directly asking about intentions of participation and intentions to express or generate support for the campaign (for items see Appendix).

### Analytic Procedure

Data analysis comprised of two parts. First, we conducted a confirmatory factor analysis and tests of measurement invariance following the procedures outlined by Vandenberg and Lance (2000) to check whether the theorized structure of the three variables derived from the needs-based model would fit our current data. Second, we tested the indirect effects of gender system justification on support for the #MeToo campaign mediated by the variables of the needs-based model for men and women separately in each national subsamples using Process macro (Hayes, 2013).

Additionally, we reran all mediation analyses with actual behaviors controlled for, and report changes in the patterns of regression weights in the controlled models to investigate whether the identified effects are simply due to justification of actual behavior (pro- or contra the campaign). We also compared model fit information of the original model (with the direct path between gender system justification and the dependent variable removed) with support for #MeToo and the variables of the needs-based model reversed. We conducted this analysis in order to offer indirect empirical support for the sequence of effects, and to rule out the possibility that support for the #MeToo campaign led to the perception that the campaign can fulfill power and moral needs, rather than what we originally assumed that the perception of the campaign fulfilling these needs predicts support for it. Both of these additional analyses were conducted using AMOS (Arbuckle, 2011) and are presented in the Supplementary Material.

### Results

#### Factor Analyses

The confirmatory factor analysis of the psychological needs items underlined that for both men and women the expected three-factor solution shows the best fit to the data, significantly better than the one- or two-factor models in all three samples (see Table 2) distinguishing between empowerment, moral reputation and moral improvement. Considering that we tested culturally embedded psychological constructs, this method did not guarantee equivalence across the samples (see van der Vijver and Tanzer, 1997). Although the three-factor model was the best factor structure for both men and women, different covariances were needed to achieve the best model fit as specified in Table 2. Therefore, we can assume structural equivalence of the scales, but not further levels of measurement invariance. The lack of invariance may very well stem from the different meaning associated with the reversed wording of the scales for men and women that we will explain in the Discussion. For this reason, we refrained from direct comparison of the data of men and women, but treat the variables as reflecting the same three underlying constructs of the needs-based model.

**TABLE 2:** Fit indices for the different factor structures of the needs-based model for women and men.

**Model**	**χ^2^**	**df**	**Δχ^2^ (compared to the 3-factor model)**	**p**	**RMSEA**	**CFI**	**TLI**	**SRMR**
**Hungarian women**								
1 factor	8291.47	35	5958.86	< 0.001	0.186 [0.18;0.19]	0.607	0.494	0.107
2 factors	5994.50	34	3661.89	< 0.001	0.160 [0.16;0.16]	0.716	0.624	0.078
3 factors	2332.61	32	–	–	0.103 [0.10;0.11]	0.890	0.846	0.046
3 factors with covariances	196.89	30	2135.72	< 0.001	0.029 [0.03;0.03]	0.992	0.988	0.022
**Hungarian men**								
1 factor	4858.88	35	1213.33	< 0.001	0.200 [0.20;0.21]	0.703	0.618	0.116
2 factors	3954.16	34	308.61	< 0.001	0.183 [0.18;0.19]	0.759	0.681	0.122
3 factors	3645.55	34	–	–	0.176 [0.17;0.18]	0.778	0.706	0.221
3 factors with covariances	393.78	29	3251.77	< 0.001	0.061 [0.06;0.07]	0.978	0.965	0.043
**Israeli women**								
1 factor	312.47	35	167.06	< 0.001	0.189 [0.17; 21]	0.580	0.460	0.124
2 factors	277.50	34	132.09	< 0.001	0.180 [0.16;. 20]	0.631	0.512	0.075
3 factors	145.41	32	–		0.126 [0.11;0.15]	0.828	0.759	0.062
3 factors with covariances	52.10	29	93.31	< 0.001	0.060 [0.03; 09]	0.965	0.946	0.041
**Israeli men**								
1 factor	221.16	35	141.49	< 0.001	0.201 [18; 23]	0.693	0.606	0.132
2 factors	160.32	34	80.75	< 0.001	0.168 [14; 19]	0.792	0.725	0.117
3 factors	79.57	32	–		0.106 [0.08; 14]	0.922	0.890	0.076
3 factors with covariances	50.18	30	29.39	< 0.001	0.071 [0.03;0.10]	0.967	0.950	0.060
**German women**								
1 factor	461.97	35	369.29	< 0.001	0.208 [0.19;0.22]	0.543	0.412	0.143
2 factors	253.83	34	161.15	< 0.001	0.151 [0.13;0.17]	0.765	0.688	0.073
3 factors	92.68	32	–		0.082 [0.06; 10]	0.935	0.909	0.042
3 factors with covariances	63.23	31	29.45	< 0.001	0.061 [0.04;0.08]	0.965	0.950	0.061
**German men**								
1 factor	288.32	35	142.23	< 0.001	0.236 [0.21;0.26]	0.548	0.419	0.138
2 factors	254.04	34	107.95	< 0.001	0.223 [0.20;0.25]	0.608	0.481	0.137
3 factors	146.09	32	–		0.166 [0.14;0.19]	0.797	0.714	0.109
3 factors with covariances	42.59	28	103.5	< 0.001	0.063 [0.02;0.10]	0.974	0.958	0.085

*In the Hungarian sample, for women covariances were created between empowerment 2 and 3, and moral reputation 2 and 3. For men between empowerment 2 and 3, 1 and 4, and moral improvement 1 and 2. In the Israeli sample for women covariances were created between empowerment 1 and 2, 2 and 4, and moral reputation 2 and 3 and for men between empowerment 2 and 4, and 1 and 3. In the German sample for women covariances were created between empowerment 2 and 4 and for men between empowerment 1 and 4, 1 and 2, moral improvement 1 and 2, and moral reputation 1 and 3.*

#### Descriptive Statistics

Descriptive statistics were calculated for the full sample, and for men and women separately, and shown in Table 3. Women supported the campaign more than men, and gender system justification scores were higher among men than women (in line with previous research e.g., Hässler et al., 2019). Consistent with the logic of the needs-based model, means of empowerment on the women’s scale were higher than the means on the men’s scale in all samples, while on both morality-related scales, men scored higher than women.

**TABLE 3:** Mean and standard deviation scores on all the study variables and a comparison of men and women.

	**α**	**Total *M* (SD)**	**Men *M* (SD)**	**Women *M* (SD)**	***T***	***p***
**Hungarian sample**						
Support for #MeToo	0.86	4.33 (1.62)	3.61 (1.70)	4.69 (1.45)	−31.80	< 0.001
Gender system justification	0.76	3.69 (1.10)	4.29 (1.13)	3.39 (0.96)	39.98	< 0.001
Empowerment	W:0.86	5.02 (1.48)	3.93 (1.47)	5.56 (1.15)		
	M:0.80					
Moral reputation	W:0.87	2.70 (1.73)	3.88 (1.96)	2.11 (1.23)		
	M:0.94					
Moral improvement	W:0.86	2.63 (1.51)	3.48 (1.57)	2.20 (1.28)		
	M:0.79					
**Israeli sample**						
Support for #MeToo	0.79	4.62 (1.43)	4.12 (1.56)	4.92 (1.26)	−5.26	< 0.001
Gender system justification	0.88	3.35 (1.33)	4.08 (1.40)	2.91 (1.07)	8.84	< 0.001
Empowerment	W:0.89	4.94 (1.62)	3.73 (1.37)	5.65 (1.29)		
	M:0.75					
Moral reputation	W:0.80	2.57 (1.82)	3.98 (1.87)	1.74 (1.16)		
	M:0.95					
Moral improvement	W:0.79	2.82 (1.99)	4.82 (1.51)	1.64 (1.09)		
	M:0.79					
**German sample**						
Support for #MeToo	0.83	4.13 (1.49)	3.34 (1.60)	4.50 (1.30)	−7.84	< 0.001
Gender system justification	0.86	3.73 (1.21)	4.50 (1.29)	3.44 (1.05)	7.81	< 0.001
Empowerment	W:0.85	5.07 (1.30)	4.14 (1.24)	5.49 (1.09)		
	M:0.75					
Moral reputation	W:0.79	2.60 (1.54)	3.75 (1.58)	2.08 (1.21)		
	M:0.88					
Moral improvement	W:0.78	2.58 (1.52)	3.72 (1.55)	2.06 (1.18)		
	M:0.88					

#### Correlations and Hypothesis Testing for the Subsamples of Women

Correlations (shown in Table 4) suggested that support for the campaign was negatively associated with gender system justification in all subsamples, and strongly positively associated with perception of the campaign as empowering. We also found an association between moral reputation, moral improvement and support for the campaign, but the association for both variables was negative in all subsamples. This means that those who felt that the campaign was an opportunity for moral improvement toward men (based on feeling guilty about women’s behavior in connection with sexual harassment and the campaign), as well as those who were concerned about the moral reputation of women in connection with the campaign supported the campaign less. In line with this, correlations between gender system justification and the two moral variables indicated a similar pattern. We found that higher system justification was associated with more concern about women’s moral reputation within the campaign and with the belief that the campaign offered an opportunity for women to show moral improvement.

**TABLE 4:** Correlations between the study variables on the subsamples of men and women.

	**1.**	**2.**	**3.**	**4.**	**5.**
**Hungarian men and women**					
1. Support #MeToo	−	−0.48***	0.61***	−0.59***	0.56***
2. Gender system justification	−0.40***	−	−0.48***	0.51***	−0.51***
3. Empowerment	0.63***	−0.36***	−	−0.71***	0.53***
4. Moral reputation	−0.35***	0.24***	−0.53***	−	−0.47***
5. Moral improvement	−0.34***	0.39***	−0.46***	0.53***	−
**Israeli men and women**					
1. Support #MeToo	−	−0.40***	0.57***	−0.51***	0.55***
2. Gender system justification	−0.34***	−	−0.35***	0.52***	−0.41***
3. Empowerment	0.66***	−0.37***	−	−0.56***	0.46***
4. Moral reputation	−0.34***	0.21***	−0.47***	−	−0.47***
5. Moral improvement	−0.36***	0.34***	−0.43***	0.49***	−
**German men and women**					
1. Support #MeToo	−	−0.59***	0.40***	−0.52***	0.59***
2. Gender system justification	−0.44***	−	−0.44***	0.59***	−0.53***
3. Empowerment	0.53***	−0.38***	−	−0.57***	0.38***
4. Moral reputation	−0.21***	0.17***	−0.36***	−	−0.39***
5. Moral behavior	−0.20***	0.25***	−0.34***	0.46***	−

Values for men are shown above the diagonal, and values for women are shown below. ***p < 0.001.

To test our hypotheses about indirect effects, confidence intervals were calculated using bootstrapping with 5,000 re-samples (Hayes, 2013) in each national sample, resulting in three models for women. Gender system justification was entered in the model as the input variable, the three variables related to the needs-based model were mediators, and support for the #MeToo was tested as the output variable. Information about the models are presented in Table 5 and the models are visually presented in Figure 1.

**TABLE 5:** Information about the mediation analysis among men and women participants in all three subsamples.

	**Coefficient**	**SE**	***t***	**LLCI**	**ULCI**	***p***
**Hungarian women**						
GSJ Empowerment	–0.44	0.01	–32.26	–0.46	–0.41	< 0.001
GSJ Moral Improvement	0.52	0.02	35.25	0.49	0.55	< 0.001
GSJ Moral reputations	0.31	0.02	20.36	0.28	0.34	< 0.001
**Outcome: support for #MeToo**						
Empowerment	0.70	0.01	48.70	0.67	0.73	< 0.001
Moral Improvement	–0.01	0.01	–0.82	–0.04	0.02	0.410
Moral Reputation	–0.01	0.01	–0.53	–0.03	0.02	0.594
GSJ (direct effect)	–0.29	0.02	–18.49	–0.32	–0.26	< 0.001
GSJ (total effect)	–0.60	0.02	–35.62	–0.57	–0.41	< 0.001
**Indirect effects of gender system justification on support (with Bootstrapping)**						
Empowerment	–0.31	0.01		–0.34	–0.29	
Moral Improvement	–0.01	0.01		–0.33	–0.28	
Moral Reputation	–0.01	0.01		–0.02	0.01	
**Israeli women**						
GSJ Empowerment	–0.45	0.08	–5.97	6.51	7.43	< 0.001
GSJ Moral Improvement	0.35	0.06	5.38	0.22	0.47	< 0.001
GSJ Moral reputations	0.23	0.07	3.19	0.087	0.37	0.002
**Outcome: support for #MeToo**						
Empowerment	0.57	0.06	9.47	0.45	0.69	< 0.001
Moral Improvement	–0.09	0.07	–1.21	–0.23	0.054	0.227
Moral Reputation	–0.01	0.07	–0.15	–0.14	0.12	0.881
GSJ (direct effect)	–0.11	0.07	–1.67	–0.24	0.02	0.097
GSJ (total effect)	–0.40	0.08	–5.34	–0.54	–0.25	< 0.001
**Indirect effects of gender system justification on support (with Bootstrapping)**						
Empowerment	–0.26	0.06		–0.39	–0.15	
Moral Improvement	–0.03	0.03		–0.09	0.04	
Moral Reputation	–0.01	0.02		–0.04	0.04	
**German women**						
GSJ Empowerment	–0.40	0.06	–6.91	–0.51	–0.28	< 0.001
GSJ Moral Improvement	0.28	0.06	4.24	0.15	0.40	< 0.001
GSJ Moral reputations	0.20	0.07	2.90	0.06	0.33	0.004
**Outcome: support for #MeToo**						
Empowerment	0.50	0.07	7.43	0.37	0.63	< 0.001
Moral Improvement	0.02	0.06	0.39	–0.10	0.15	0.698
Moral Reputation	–0.02	0.06	–0.37	–0.14	0.10	0.714
GSJ (direct effect)	–0.35	0.07	–5.25	–0.47	–0.22	< 0.001
GSJ (total effect)	–0.54	0.07	–8.13	–0.67	–0.41	< 0.001
**Indirect effects of gender system justification on support (with Bootstrapping)**						
Empowerment	–0.20	0.05		–0.29	–0.10	
Moral Improvement	0.01	0.02		–0.03	0.04	
Moral Reputation	–0.01	0.01		–0.03	0.02	
**Hungarian men**						
GSJ Empowerment	–0.63	0.02	–32.08	–0.66	–0.59	< 0.001
GSJ Moral Improvement	–0.72	0.02	–35.04	–0.76	–0.68	< 0.001
GSJ Moral reputations	0.89	0.03	35.01	0.84	0.94	< 0.001
**Outcome: support for #MeToo**						
Empowerment	0.31	0.02	14.37	0.26	0.35	< 0.001
Moral Improvement	0.29	0.02	17.33	0.25	0.32	< 0.001
Moral Reputation	–0.19	0.02	–12.09	–0.22	–0.16	< 0.001
GSJ (direct effect)	–0.17	0.02	–7.15	–0.21	–0.12	< 0.001
GSJ (total effect)	–0.73	0.02	–32.42	–0.78	–0.69	< 0.001
**Indirect effects of gender system justification on support (with Bootstrapping)**						
Empowerment	–0.19	0.02		–0.22	–0.16	
Moral Improvement	–0.21	0.02		–0.24	–0.18	
Moral Reputation	–0.17	0.02		–0.20	–0.14	
**Israeli men**						
GSJ Empowerment	–0.35	0.08	–4.31	–0.51	–0.19	< 0.001
GSJ Moral Improvement	–0.44	0.09	–5.16	–0.62	–0.27	< 0.001
GSJ Moral reputations	0.70	0.10	7.00	0.50	0.90	< 0.001
**Outcome: support for #MeToo**						
Empowerment	0.37	0.09	3.95	0.18	0.55	< 0.001
Moral Improvement	0.31	0.08	3.81	0.15	0.47	< 0.001
Moral Reputation	–0.12	0.07	–1.63	–0.27	0.03	0.105
GSJ (direct effect)	–0.10	0.09	–1.10	–0.27	0.08	0.273
GSJ (total effect)	–0.45	0.09	–1.97	–0.62	–0.27	< 0.001
**Indirect effects of gender system justification on support (with Bootstrapping)**						
Empowerment	–0.13	0.05		–0.24	–0.05	
Moral Improvement	–0.14	0.05		–0.24	–0.06	
Moral Reputation	–0.08	0.06		–0.20	0.05	
**German men**						
GSJ Empowerment	–0.43	0.08	–5.59	–0.58	–0.28	< 0.001
GSJ Moral Improvement	–0.64	0.09	–7.14	–0.82	–0.47	< 0.001
GSJ Moral reputations	0.72	0.09	8.16	0.54	0.89	< 0.001
**Outcome: support for #MeToo**						
Empowerment	0.04	0.10	0.33	–0.17	0.24	0.735
Moral Improvement	0.37	0.08	4.64	0.21	0.52	< 0.001
Moral Reputation	–0.21	0.09	–2.39	–0.39	–0.04	0.019
GSJ (direct effect)	–0.33	0.11	–3.01	–0.54	–0.11	0.003
GSJ (total effect)	–0.73	0.09	–8.25	–0.90	–0.55	< 0.001
**Indirect effects of gender system justification on support (with Bootstrapping)**						
Empowerment	–0.02	0.05		–0.12	0.08	
Moral Improvement	–0.24	0.06		–0.36	–0.13	
Moral Reputation	–0.15	0.06		–0.27	–0.04	

In all three samples, as expected, we found that empowerment mediated the connection between gender system justification and support, suggesting that women with lower gender system-justification considered the campaign more empowering which in turn predicted higher support for the campaign. For Hungarian women the explained variance was 43% (*R*^2^ = 0.43, *F*(4,6804) = 1290.95, *p* < 0.001), for Isreali women it was 44% (*R*^2^ = 0.44, *F*(4,217) = 43.31, *p* < 0.001), and for German women it was 34% (*R*^2^ = 0.34, *F*(4,278) = 36.05, *p* < 0.001).

In two additional analyses, we tested whether the pattern of connections remain the same after controlling for actual participation in the campaign in order to rule out that support was merely a justification of either actually participating in the campaign or supporting the campaign in online posts and comments, or on the contrary, lack of support for the campaign was justification for criticizing the campaign online. We found no changes in the patterns after controlling for these affects, however, all regression weights became somewhat smaller. Secondly, we reran all analyses by reversing the order of the variables of the needs-based model and support for the campaign in the model and by removing the direct path between gender system justification and the dependent variable (which already resulted in deteriorated models). In the Hungarian and Israeli samples, we found substantial decrease in model fit, however, in case of the German subsample, the reversed model showed actually better fit than the original. Results of the controlled models and information about model fit changes in the reversed models are presented in the Supplementary Material.

#### Correlations and Hypothesis Testing for the Subsamples of Men

As shown in Table 4, gender system justification was negatively associated with support for the campaign similarly to the women samples, and positively associated with empowerment. Perceptions of the campaign as an opportunity for moral improvement and as a threat to moral reputation were strongly associated with support for the campaign in the expected direction: moral reputation negatively and moral improvement positively. A further analysis of correlations between the study variables showed that gender system justification was strongly positively associated with moral reputation and strongly negatively with moral improvement in all samples, suggesting that those endorsed more system critical ideas about gender relations felt that the campaign was an opportunity for moral improvement stemming from guilt, but were not concerned about the campaign staining the moral reputation of men.

Results of the mediation analyses for men were less similar across the samples than for women (see Table 5 and Figure 1). In the Hungarian sample, explained variance was 49% (*R*^2^ = 0.49, *F*(4,3424) = 829.16, *p* < 0.001) and all three psychological needs variables mediated the connection between gender system justification and support for #MeToo. This is less surprising, considering the large sample size, however, power-related needs were even stronger predictors than either of the moral needs. Similarly, in the Israeli sample of men where explained variance was 46% (*R*^2^ = 0.46, *F*(4,127) = 26.87, *p* < 0.001), power-related needs were the strongest predictors of support, whereas moral reputation was not a significant predictor here. This means that male participants (in these two contexts) who were concerned about potential loss of power due to the campaign, supported it less. However, the need for empowerment was not a significant mediator of the effect of gender system justification on support for the campaign in the German sample, where only moral reputation and improvement mediated this connection. Explained variance was 48% here (*R*^2^ = 0.48, *F*(4,125) = 29.36, *p* < 0.001).

We ran the two additional analyses for men too. Again, we identified a similar pattern when actual behavior was controlled in the models, but effects were somewhat smaller. Reversing the order of the variables of the needs-based model and support for #MeToo led to worse model fit in all three samples of men. Results of the controlled models and information about model fit changes in the reversed models are presented in the Supplementary Material.

## Discussion

Relying on an unusually large sample in the case of Hungary, and on follow-up surveys in two other national contexts, Israel and Germany, we investigated whether support for #MeToo can be understood by looking at women’s and men’s satisfaction of (or threat to) different psychological needs, as members of victim and perpetrator groups, respectively. Our hypothesis was based on the assumption that sexual harassment is not an interpersonal issue, but it is embedded in gender relations in society (Searles, 1995) as the campaign itself suggested. In support of our hypothesis, we found that higher gender system justification predicted less support for the campaign among both women and men. This suggests that the campaign was more positively evaluated by those who were generally more critical of the existing gender arrangements – similar to patterns observed in research on the predictors of collective action tendencies related to gender issues (Calogero, 2017) and sexual violence (Chapleau and Oswald, 2014). These findings demonstrate, in line with previous research on the #MeToo campaign (Kunst et al., 2018), that support for the campaign should be evaluated as an intergroup issue related to gender relations.

Furthermore, based on the needs-based model (Shnabel et al., 2009), we hypothesized that for women the debate about sexual harassment – as it is embedded in the broader context of gender relations – would be perceived through the prism of empowerment (i.e., it would be conceptualized as a struggle about control and agency). For men, by contrast, it would be mainly perceived through the prism of morality (i.e., it would be conceptualized as a struggle about who is “good” and who is “bad”), as well as by their fear to lose status and privilege. Our findings supported the idea that women who were more critical of the existing gender arrangements viewed the campaign as more empowering, which in turn predicted their support for it. This connection (between system justification and campaign support) was not mediated by concerns about moral reputation or by the opportunity for moral improvement. This suggests that for women, the satisfaction of (or threat to) moral needs was irrelevant for whether or not attitudes toward gender relations would translate into support of (or opposition to) the #MeToo campaign.

For men, supporting our hypotheses, concerns for moral reputation negatively (except in the Israeli sample) and perceived opportunity for moral improvement positively predicted support for the campaign – mediating the effect of gender system justification on campaign support. These results are consistent with previous findings, observed in contexts of direct violence (e.g., abuse of war prisoners), that guilt and concerns about the ingroup’s moral conduct are associated with positive outgroup attitudes, whereas concerns about the ingroup’s moral reputation are associated with negative outgroup attitudes (Allpress et al., 2014). Also, the finding that the more men viewed the campaign as an opportunity for their ingroup to improve its moral conduct the more they supported it, is consistent with the theorizing that for members of perpetrator groups the opportunity for satisfying moral needs increases reconciliation efforts (Shnabel et al., 2009). It is also consistent with findings that system-critical attitudes can be predictors of collective action intentions among allies who are striving to improve their moral identity by behaving more morally (Hopkins et al., 2007; Brambilla et al., 2013).

In line with some previous findings about men’s status threat (see Hässler et al., 2019) and our hypothesis, men’s perceptions of the campaign as disempowering was negatively correlated with support for the campaign in all samples and it also mediated the connection between gender system justification and opposition for the campaign among men in the Hungarian and Israeli samples, but not in the German sample. In fact, in these two samples, support for the campaign was more strongly predicted by a threat to their ingroup’s status and power than by the satisfaction or thwarting of either of the moral needs. The link between men’s concerns about gender power relations and their attitudes toward sexual harassment are in line with previous findings. For example, men are more likely to engage in sexual harassment when their masculine identity (Hitlan et al., 2009) or social status is threatened (Berdahl, 2007), when they feel that the legitimacy and distinctiveness of the current *status quo* is under threat (Maass et al., 2003), when they are afraid to be perceived as incompetent (Halper and Rios, 2019), and toward women who express egalitarian, rather than traditional gender-role attitudes (Dall’Ara and Maass, 1999). Although women identifying as feminists experience as much sexual harassment as other women, women engaging in feminist activism suffered more gender-based harassment (Holland and Cortina, 2013). This finding also fits with recent research suggesting that advantaged group members’ opposition to policies that empower disadvantaged groups stems both from moral motivations (i.e., the wish to defend their ingroup’s positive moral identity, in the face of accusation that they enjoy unearned privilege), and from their wish to maintain power (Kahalon et al., 2019).

However, contrary to our hypothesis, this connection was not present in the German subsample of men, where only moral needs mediated the connection between gender system justification and support for #MeToo. This finding, which is consistent with our original theorizing based on the needs-based model, may stem from the different cultural contexts of the data collection in terms of gender (in)equality. It seems that in the German context men’s support for #MeToo was not undermined by men’s power related concerns. This finding is consistent with research suggesting that gender equality and attitudes toward sexual harassment are interconnected (Chapleau and Oswald, 2014). Consequently, in the German context which is characterized by greater gender equality compared to the other two countries, men have less privilege to lose and may consider gender equality as beneficial for both men and women.

We wanted to rule out the possibility that the connection between gender system justification and support for the campaign was mediated by the psychological needs of the needs-based model merely as a justification for actual participation in the campaign either in the form of posting one’s own story using the #MeToo hashtag or posting a supportive or critical comment about the campaign online. Therefore, we reran all analyses with these behaviors controlled for. In all six samples we found models that were highly similar to the original one, suggesting that the connection was not simply the result of justifying their own behavior. For the purpose of controlling whether our assumptions about the sequence of predictions was supported by the data, we also tested the models by reversing the order of support for #MeToo and psychological needs. In five out of six models, model fit decreased compared to the original models supporting our original hypothesis about the order of effects. However, among German women, we found an increase in model fit, suggesting the possibility that within this subsample higher support for the campaign was the reason participants perceived the campaign as a source of empowerment (e.g., as a form of justifying their support), rather than the perception of the campaign as an empowering movement led to its support.

Finally, we need to reflect on the finding that the correlations between moral improvement and support for #MeToo among men and women were in opposite directions. Women who viewed the campaign as an opportunity for moral improvement showed less support for the campaign, whereas the opposite association was observed among men. We interpret these findings as suggesting that as members of the perpetrator group, men may seek ways to improve their moral identity, and if this campaign is perceived to offer this opportunity, they show higher support. However, women who viewed the campaign as an opportunity to improve their moral behavior likely believed that women should stop seducing men into sexual harassment or refrain from falsely accusing men in the campaign (in line with the myth that women lie about rape, see Lonsway and Fitzgerald, 1994 and with the hostile sexist belief that women use their sexuality to exploit men; Glick and Fiske, 2001). As a result, they view the campaign as an opportunity for moral improvement (e.g., if it leads women to stop seducing innocent men). Previous research has revealed that due to stigma internalization processes, members of disadvantaged groups (e.g., sexual minorities; Górska et al., 2017), including women, may adopt negative views about their ingroup’s morality; alternatively, they may be aware of this stigma and try to refute it. For example, women’s engagement in competitive victimhood (i.e., effort to prove that their ingroup suffers more injustice than men) were partially driven by their need to defend their ingroup’s moral reputation (Sullivan et al., 2012; Kahalon et al., 2019). Similar processes of stigma internalization have been observed in sexual minorities: respondents who were high in system justification wished that their ingroup would behave more morally (Hässler et al., 2019) because they adopted the view of sexual minorities as morally deviant (Herek and McLemore, 2013). Therefore, the different results may stem from the different associations with the items measuring moral improvement for men and women and therefore not entirely surprising or contradictory to previous research. However, they clearly demonstrate that these victim-blaming beliefs among women can become an obstacle for women to support social change action to reduce sexual harassment.

### Limitations

Our research was conducted in the context of a real-life campaign while the topic was timely and widely discussed. This overwhelming interest in the topic allowed us to collect an unusually large sample in Hungary. However, despite the large sample size, it was not representative of the Hungarian population. Therefore, conclusions regarding the Hungarian society in general, such as women being more supportive of the campaign than men, should be drawn cautiously (as for the Israeli and German samples as well). Also, while problems related to small sample sizes have been extensively discussed in social psychology and other disciplines (e.g., Wolf et al., 2013), large sample sizes can create statistical challenges too. For example, such a large sample can enlarge biases embedded in the sampling method (Kaplan et al., 2014). Specifically, our call may have attracted participants in Hungary with a stronger opinion about the campaign either pro or contra, resulting in stronger connections between the variables than we would have found among the general population using probability sampling. However, the similarity of the results across the contexts suggests otherwise and strengthens the study’s conclusions.

The cross-sectional data of the current research cannot offer evidence for causal connection between the variables. In order to offer indirect support for the order of the effects, we reran the models with the variables in a reversed order which generally supported our theorization about causality, however, it remains plausible that the connection between these variables is circular, rather than one-directional, and that besides the perception of needs-satisfaction leading to higher campaign support, higher campaign support leads to higher perception of the campaign as fulfilling these psychological needs. For example, women who support this campaign (e.g., due to their feminist identification, Kunst et al., 2018) may in turn perceive it as more empowering (as a form of *post hoc* justification) as we identified it among German women. Future research, relying on longitudinal data could offer a definite answer to that.

Also, as the needs-based model captures the psychological needs of perpetrators and victims in dyadic conflicts, we could not integrate the perspective of respondents outside the gender binary. We acknowledge this as a shortcoming of our research, especially considering the relevance of sexual harassment in the lives of sexual minorities (Stotzer, 2009). Future research should capture the psychological needs that are specific for these groups in predicting the support for social movements in the area of sexual harassment. Furthermore, respondents completed the questionnaire based on perceptions of need satisfaction of their own gender ingroup in the context of the #MeToo campaign, while we can expect that perceived need satisfaction of the other gender group may have affected support too. For example, men who considered the campaign disempowering to women and women who considered the campaign as unjustifiably staining men’s moral reputation may have shown less support. Testing these connections is an interesting direction for future research.

Finally, collecting additional data in Israel and Germany was intended to increase the validity of our conclusions based on the results from Hungary. In collecting these two additional samples, our main focus remained the analysis of psychological motivations in supporting the campaign, rather than offering cross-cultural comparisons or a broader sociological understanding of support for the campaign. This approach allowed us to draw conclusions regarding some of the psychological mechanisms that motivate people to engage in collective action in support of social change in connection with sexual harassment. However, an analysis of demographic variables and political ideology would be needed to offer a description of the level of campaign support across different segments of society.

## Conclusions and Practical Implications

Previous studies (e.g., Becker and Barreto, 2014) have outlined the different obstacles men and women have in recognizing gender inequality and joining social change efforts of feminist movements. In line with this, our research corroborated the importance of awareness of structural injustice by highlighting the connection between support for #MeToo and low gender system justification and sexual harassment (similarly to the findings of Kunst et al., 2018). We further showed that these general attitudes, which influence the extent to which men and women view of their ingroup as a perpetrator and a victim group (Hässler et al., 2019), can translate into support for the campaign if it seemed to fulfill the power and morality needs resulting from these social roles. These findings point to a potential source of misunderstandings (Demoulin et al., 2009) between men and women about the phenomenon of sexual harassment, because besides a power struggle *per se*; such that (some) women want to gain power through the campaign, whereas (some) men are afraid to lose power through it, women and men interpret this phenomenon through different prisms. Women who are more critical of gender status differences view the problem of sexual harassment and the related campaign through the lens of empowerment, whereas men with similarly critical attitudes perceive it also through the lens of morality.

While the campaign was undoubtedly extremely successful in reaching millions of people globally, our findings allow to identify the reasons why some men and women feel reluctant to support it. These findings provide insights as to which communication strategies can effectively promote support for the struggle against sexual harassments, which can be taken into account when mobilizing men and women for mass protests, as well as when designing intervention programs or educational preventions challenging gender relations.

For example, the finding that system justification is negatively and directly related to less campaign support among both women and men suggests that to increase support among high system justifiers it may be beneficial to use system-affirmation strategies (e.g., Brescoll et al., 2013). For example, anti-harassment activists may highlight that nowadays women receive treatment that is fairer than in any other historical period, yet eliminating sexual harassment is required to further strengthen the existing system. Such strategies, which highlight the positive aspects of the system rather than condemning it, may satisfy high system-justifiers’ strong need to feel that the existing system is legitimate and increase their support for fighting against sexual harassment.

Specifically with regards to men, our findings suggest that presenting current awareness of the nature and prevalence of sexual harassment as a unique opportunity for repentance, which can restore harmonious gender relations, should increase men’s support for the struggle against sexual harassment. Moreover, the finding that men’s moral reputation concerns are associated with opposition to the campaign implies that men’s defensiveness and consequent opposition can be reduced through the affirmation of their ingroup’s morality, for example, by highlighting that harassment is not a typical male behavior as most men treat women with respect. Similar moral affirmation strategies were found to be effective in increasing White Americans’ willingness to address grievances of Black Americans (Ditlmann et al., 2017). In the context of gender relations, the positive portrayal of feminist men increased men’s solidarity with women that in turn translated into collective action intentions (Wiley et al., 2013).

Finally, the finding that men’s support for the campaign in Hungary and in Israel was negatively influenced by their power concerns implies that one strategy to increase support, at least in countries characterized by relatively low gender equality, would be to counter perceptions of gender relations as a zero-sum situation. That is, use communication strategies that argue that empowering women does not mean disempowering men, as both groups have common interests. Similar strategies effectively increased readiness to support policies to empower immigrants among host members (Esses et al., 1998).

As for women, our findings suggest that their support for the struggle against sexual harassment would increase if they believe that it strengthens their ingroup. Hence, a possible route to increase support would be conveying the message that receiving acknowledgment of one’s victimization does not imply that one is weak and humiliated, rather, such acknowledgment is the necessary first step toward greater agency (such steps are described in the literature on the effects of apologies for example, see Hornsey et al., 2015). Moreover, the negative association between women’s view of the campaign as an opportunity for moral improvement and support for the campaign implies that at least some women have a preference for less confrontational campaigns (e.g., as they believe that women too should improve their moral conduct). Such campaigns against sexual harassment could mobilize men and women by emphasizing shared ideals and values rather than intergroup differences (for a similar strategy in the context of racial relations in the United States see Ditlmann et al., 2017). In conclusion, the insights gained through our findings promote a deeper understanding of the factors that facilitate men’s and women’s support for the struggle against sexual harassment, and allow to identify strategies to remove the psychological obstacles that hinder such support.

## Data Availability Statement

The datasets for this study can be found in the Open Science Framework site at osf.io/rj94d.

## Ethics Statement

The studies involving human participants were reviewed and approved by Faculty of Education and Psychology, Eötvös Loránd University. The patients/participants provided their written informed consent to participate in this study.

## Author Contributions

AK contributed the research idea and the general design of the study and was responsible for data analysis and the preparation of the manuscript. NS contributed to the idea, the design of the study and the preparation of manuscript. BN and NL contributed to the design of the study, data collection in Hungary, data analysis, preparation of the figures and tables. MH contributed to the statistical analysis. DP and JK translated the questionnaires to Hebrew and German, respectively, and collected the data in Israel and Germany.

## Conflict of Interest

The authors declare that the research was conducted in the absence of any commercial or financial relationships that could be construed as a potential conflict of interest.

## Appendix

**Needs-Based Items**

Empowerment:

1. In the long run, campaigns like this weaken women/men as a group (reversed item).
2. In the long run, campaigns like this make women/men more vulnerable (reversed item).
3. This campaign empowers women/men.
4. This campaign increases women’s/men’s control over their lives.

Moral improvement:

1. The campaign makes me wish that women/men would treat men/women in a nicer manner.
2. The campaign makes me feel ashamed of the unfair behavior of women/men toward men/women.
3. The campaign makes me feel guilty about what men/women have to put up with due to women’s/men’s immoral behavior.

Moral reputation:

1. The campaign unjustifiably stains women’s/men’s moral reputation.
2. The campaign might unjustifiably create an image of women/men as immoral.
3. The campaign wrongfully presents women/men as bad people.

Support for #MeToo items:

1. Assuming that you were personally affected, would you share your own story within the #MeToo campaign?
2. Regardless of whether you are personally affected, would you express support for the #MeToo campaign on social media?
3. Would you express support for the campaign outside social media (for example to your friends, colleagues, and acquaintances)?
4. Would you encourage others to share their own personal stories within the campaign?
