# Supplementary material for: A Needs-Based Support for #MeToo: Power and Morality Needs Shape Women’s and Men’s Support of the Campaign
Source: Front Psychol. 2020 Mar 31;11:593. doi: 10.3389/fpsyg.2020.00593 (PMC7136498; doi:10.3389/fpsyg.2020.00593)
Supplement: Supplementary file 1 [file Table_1.DOCX]

*S1. Information about the mediation analysis among men and women participants in all three subsamples with own posting behavior (own story, supportive or critical comment) controlled for in the models.*

|  | Coefficient | SE | LLCI | ULCI | p |
| --- | --- | --- | --- | --- | --- |
| **Hungarian women** | | | | | |
| GSJ🡪Empowerment | -0.32 | 0.02 | -0.35 | -0.30 | <.001 |
| GSJ🡪Moral Improvement | 0.46 | 0.02 | 0.42 | 0.49 | <.001 |
| GSJ🡪 Moral reputations | 0.21 | 0.01 | 0.18 | 0.23 | <.001 |
| Outcome: support for #MeToo | | | | | |
| Empowerment | 0.60 | 0.01 | 0.57 | 0.63 | <.001 |
| Moral Improvement | -0.01 | 0.01 | -0.04 | 0.02 | .652 |
| Moral Reputation | -0.00 | 0.01 | -0.02 | 0.02 | .748 |
| GSJ (direct effect) | -0.20 | 0.02 | -0.23 | -0.17 | <.001 |
| GSJ (total effect) | -0.40 | 0.02 | -0.43 | -0.37 | <.001 |
| Indirect effects of gender system justification on support (with Bootstrapping) | | | | | |
| Empowerment | -0.20 | 0.01 | -0.22 | -0.18 |  |
| Moral Improvement | -0.01 | 0.01 | -0.02 | 0.01 |  |
| Moral Reputation | 0.00 | 0.00 | -0.01 | 0.01 |  |
| **Israeli women** | | | | | |
| GSJ🡪Empowerment | -0.36 | 0.02 | -0.41 | -0.32 | <.001 |
| GSJ🡪Moral Improvement | 0.30 | 0.02 | 0.26 | 0.34 | <.001 |
| GSJ🡪 Moral reputations | 0.21 | 0.01 | 0.18 | 0.23 | <.001 |
| Outcome: support for #MeToo | | | | | |
| Empowerment | 0.52 | 0.02 | 0.48 | 0.56 | <.001 |
| Moral Improvement | -0.06 | 0.03 | -0.11 | -0.01 | .013 |
| Moral Reputation | -0.00 | 0.01 | -0.02 | 0.02 | .748 |
| GSJ (direct effect) | -0.04 | 0.02 | -0.08 | -0.00 | .043 |
| GSJ (total effect) | -0.24 | 0.02 | -0.29 | -0.20 | <.001 |
| Indirect effects of gender system justification on support (with Bootstrapping) | | | | | |
| Empowerment | -0.18 | 0.02 | -0.21 | -0.15 |  |
| Moral Improvement | -0.02 | 0.01 | -0.04 | 0.00 |  |
| Moral Reputation | 0.00 | 0.00 | -0.01 | 0.01 |  |
| **German women** | | | | | |
| GSJ🡪Empowerment | -0.35 | 0.02 | -0.40 | -0.30 | <.001 |
| GSJ🡪Moral Improvement | 0.29 | 0.02 | 0.25 | 0.33 | <.001 |
| GSJ🡪 Moral reputations | 0.21 | 0.07 | 0.18 | 0.23 | <.001 |
| Outcome: support for #MeToo | | | | | |
| Empowerment | 0.42 | 0.02 | 0.38 | 0.46 | <.001 |
| Moral Improvement | -0.01 | 0.02 | -0.04 | 0.03 | .595 |
| Moral Reputation | -0.00 | 0.01 | -0.02 | 0.02 | .748 |
| GSJ (direct effect) | -0.28 | 0.02 | -0.32 | -0.25 | <.001 |
| GSJ (total effect) | -0.43 | 0.02 | -0.48 | -0.40 | <.001 |
| Indirect effects of gender system justification on support (with Bootstrapping) | | | | | |
| Empowerment | -0.14 | 0.01 | -0.17 | -0.12 |  |
| Moral Improvement | -0.00 | 0.01 | -0.01 | 0.01 |  |
| Moral Reputation | -0.01 | 0.00 | -0.01 | 0.00 |  |
| **Hungarian men** | | | | | |
| GSJ🡪Empowerment | -0.44 | 0.02 | -0.48 | -0.41 | <.001 |
| GSJ🡪Moral Improvement | -0.60 | 0.02 | -0.64 | -0.60 | <.001 |
| GSJ🡪 Moral reputations | 0.71 | 0.03 | -0.66 | -0.76 | <.001 |
| Outcome: support for #MeToo | | | | | |
| Empowerment | 0.26 | 0.02 | 0.27 | 0.34 | <.001 |
| Moral Improvement | 0.26 | 0.02 | 0.23 | 0.29 | <.001 |
| Moral Reputation | -0.17 | 0.02 | -0.19 | -0.14 | <.001 |
| GSJ (direct effect) | -0.12 | 0.02 | -0.16 | -0.07 | <.001 |
| GSJ (total effect) | -0.53 | 0.02 | -0.57 | -0.48 | <.001 |
| Indirect effects of gender system justification on support (with Bootstrapping) | | | | | |
| Empowerment | -0.15 | 0.01 | -0,17 | -0.12 |  |
| Moral Improvement | -0.17 | 0.01 | -0.19 | -0.14 |  |
| Moral Reputation | -0.14 | 0.02 | -0.17 | -0.11 |  |
| **Israeli men** | | | | | |
| GSJ🡪Empowerment | -0.44 | 0.02 | -0.47 | -0.41 | <.001 |
| GSJ🡪Moral Improvement | -0.38 | 0.03 | -0.44 | -0.32 | <.001 |
| GSJ🡪 Moral reputations | 0.65 | 0.03 | 0.59 | 0.71 | <.001 |
| Outcome: support for #MeToo | | | | | |
| Empowerment | 0.31 | 0.02 | 0.27 | 0.34 | <.001 |
| Moral Improvement | 0.29 | 0.03 | 0.25 | 0.35 | <.001 |
| Moral Reputation | -0.12 | 0.03 | -0.17 | 0.07 | <.001 |
| GSJ (direct effect) | -0.08 | 0.03 | -0.14 | -0.02 | .012 |
| GSJ (total effect) | -0.41 | 0.03 | -0.46 | -0.35 | <.001 |
| Indirect effects of gender system justification on support (with Bootstrapping) | | | | | |
| Empowerment | -0.11 | 0.01 | -0.14 | -0.08 |  |
| Moral Improvement | -0.09 | 0.01 | -0.12 | -0.07 |  |
| Moral Reputation | -0.06 | 0.01 | -0.09 | 0.03 |  |
| **German men** | | | | | |
| GSJ🡪Empowerment | -0.36 | 0.03 | -0.42 | -0.31 | <.001 |
| GSJ🡪Moral Improvement | -0.62 | 0.03 | -0.68 | -0.56 | <.001 |
| GSJ🡪 Moral reputations | 0.69 | 0.03 | 0.62 | 0.75 | <.001 |
| Outcome: support for #MeToo | | | | | |
| Empowerment | 0.05 | 0.03 | -0.02 | 0.11 | .151 |
| Moral Improvement | 0.34 | 0.02 | 0.30 | 0.38 | <.001 |
| Moral Reputation | -0.25 | 0.02 | -0.29 | -0.20 | <.001 |
| GSJ (direct effect) | -0.25 | 0.04 | -0.32 | -0.19 | <.001 |
| GSJ (total effect) | -0.65 | 0.03 | -0.70 | -0.59 | <.001 |
| Indirect effects of gender system justification on support (with Bootstrapping) | | | | | |
| Empowerment | -0.02 | 0.01 | -0.04 | 0.01 |  |
| Moral Improvement | -0.21 | 0.02 | -0.25 | -0.18 |  |
| Moral Reputation | -0.17 | 0.02 | -0.21 | -0.14 |  |

*S2. Information about model fit changes in the original model (direct path between gender system justification and the dependent variable removed) compared with models in which variables of the Needs-based model and support for #MeToo were reversed.*

|  | χ2 | df | CFI | RMSEA | AIC | SRMR |
| --- | --- | --- | --- | --- | --- | --- |
| **Hungarian women** | | | | | | |
| Model in original order | 338.06 | 1 | 0.967 | 0.222 | 376.06 | 0.039 |
| Model with reversed order | 674.75 | 3 | 0.935 | 0.181 | 708.75 | 0.077 |
| **Israeli women** | | | | | | |
| Model in original order | 28.28 | 1 | 0.991 | 0.111 | 66.28 | 0.02 |
| Model with reversed order | 198.5 | 3 | 0.936 | 0.171 | 232.5 | 0.73 |
| **German women** | | | | | | |
| Model in original order | 266.97 | 1 | 0.907 | 0.307 | 304.97 | 0.06 |
| Model with reversed order | 165.69 | 3 | 0.943 | 0.139 | 199.89 | 0.061 |
| **Hungarian men** | | | | | | |
| Model in original order | 49.12 | 1 | 0.994 | 0.118 | 87.12 | 0.018 |
| Model with reversed order | 689.79 | 3 | 0.908 | 0.258 | 723.79 | 0.098 |
| **Israeli men** | | | | | | |
| Model in original order | 12.52 | 1 | 0.995 | 0.093 | 50.52 | 0.015 |
| Model with reversed order | 273.02 | 3 | 0.878 | 0.261 | 307.02 | 0.101 |
| **German men** | | | | | | |
| Model in original order | 90.84 | 1 | 0.963 | 0.263 | 128.84 | 0.037 |
| Model with reversed order | 338.21 | 3 | 0.862 | 0.293 | 372.21 | 0.103 |
